# An Atypical Unfolded Protein Response in Heat Shocked Cells

**DOI:** 10.1371/journal.pone.0023512

**Published:** 2011-08-10

**Authors:** Lonneke Heldens, Sanne M. M. Hensen, Carla Onnekink, Siebe T. van Genesen, Ron P. Dirks, Nicolette H. Lubsen

**Affiliations:** Department of Biomolecular Chemistry, Radboud University Nijmegen, Nijmegen, The Netherlands; St. Georges University of London, United Kingdom

## Abstract

**Background:**

The heat shock response (HSR) and the unfolded protein response (UPR) are both activated by proteotoxic stress, although in different compartments, and share cellular resources. How these resources are allocated when both responses are active is not known. Insight in possible crosstalk will help understanding the consequences of failure of these systems in (age-related) disease.

**Results:**

In heat stressed HEK293 cells synthesis of the canonical UPR transcription factors XBP1s and ATF4 was detected as well as HSF1 independent activation of the promoters of the ER resident chaperones HSPA5 (BiP) and DNAJB9 (ERdj4). However, the heat stress activation of the DNAJB9 promoter, a XBP1s target, was not blocked in cells expressing a dominant negative IRE1α mutant, and thus did not require XBP1s. Furthermore, the DNA element required for heat stress activation of the DNAJB9 promoter is distinct from the ATF4 and ATF6 target elements; even though inhibition of eIF2α phosphorylation resulted in a decreased activation of the DNAJB9 promoter upon heat stress, suggesting a role for an eIF2α phosphorylation dependent product.

**Conclusions:**

The initial step in the UPR, synthesis of transcription factors, is activated by heat stress but the second step, transcriptional transactivation by these factors, is blocked and these pathways of the UPR are thus not productive. Expression of canonical ER chaperones is part of the response of heat stressed cells but another set of transcription factors has been recruited to regulate expression of these ER chaperones.

## Introduction

All cells contain an extensive network of chaperones to maintain proteostasis. When proteostasis is disturbed, additional chaperones are synthesized to restore protein folding or to increase removal of irreversibly unfolded proteins by targeting these for degradation. For reviews see [1]–[4]. Eukaryotic cells have two evolutionarily highly conserved systems to combat proteotoxic stress: the heat shock (HS) system and the unfolded protein response (UPR). The HS system is the major response to stress conditions in the cytosol [5], while cells respond to the accumulation of unfolded proteins in the lumen of the endoplasmic reticulum by activating the UPR.

The UPR induces a transient attenuation of protein synthesis and a transcriptional activation of genes to expand the protein-folding capacity of the ER. These responses are mediated by three ER-localized transmembrane proteins: inositol requiring 1α (IRE1 α), PKR-like endoplasmic reticulum kinase (PERK), and activating transcription factor 6 (ATF6) [6]–[9]. Under non-stressed conditions, these proteins are sequestered by the chaperone HSPA5 (BiP). Unfolded proteins in the ER compete for HSPA5 and IRE1α, PERK and ATF6 are released [10]. Activation of IRE1α results in the removal of a 26-nucleotide intron from XBP1 mRNA allowing the synthesis of the transcription factor XBP1 [11], [12]. Activation of PERK, an eIF2α kinase, leads to phosphorylation of eIF2α and thus to an overall inhibition of the initiation of protein synthesis [13]. Paradoxically, it also results in the preferential translation of some downstream ORFs, known as stress induced leaky scanning [14]. Stress induced leaky scanning is essential for the translation of the ATF4 ORF [15], [16]. In addition to ATF4 mRNA, GADD34 [17] and ATF5 ORFs [18], [19] are also subject to translational upregulation in response to eIF2α phosphorylation. GADD34 is a regulatory subunit of protein phosphatase I and mediates eIF2α -P dephosphorylation. The gene for GADD34 is also one of the targets of ATF4. GADD34 is thus part of a feedback loop [20], [21]. ATF4, together with XBP1s and ATF6, directs the transcriptional response of the UPR. The heat shock response (HSR) shows some parallels with the UPR. The HSR is mediated by a single transcription factor, heat shock factor 1 (HSF1) [22]. Like the mediators of the UPR, HSF1 is sequestered by chaperones. In unstressed cells HSF1 is in the cytoplasm in a complex containing the chaperone Hsp90. Unfolding proteins compete for Hsp90 and upon its release from the Hsp90 complex HSF1 is activated [23]–[26]. HSF1 enhances the transcription of the so-called heat shock genes, genes that encode cytoplasmic chaperones such as HSPA1A (Hsp70), DNAJB1 (Hsp40) and HSPB1 (Hsp27) [27]. Like the UPR, a heat shock also results in activation of an eIF2α kinase, in this case both PKR and HRI [28], [29]. In addition initiation of translation is inhibited through inhibition of the cap-binding complex [30]–[32].

It is likely that there is cross-talk between the HSR and the UPR. These two responses share a resource, the proteasome, which degrades both the irreversibly folded cytoplasmic and ER proteins – the latter via the (ER)-associated degradation (ERAD) pathway [33] - and they share the eIF2α kinase regulatory pathway. The HSR and the UPR also compete for resources in the, not unlikely, case that a stressor causes protein unfolding in both cellular compartments. Indeed, a heat stress has been shown to transiently induce XBP1 splicing [34] and to lead to an increase in HSPA5 and DNAJB9 (ERdj4) mRNA levels [35], both typical UPR responses. We show here that heat stress induces an UPR like response, but that this response is not productive. The activation of the HSPA5 and DNAJB9 promoters is a late response of cells recovering from heat stress and the DNAJB9 promoter is not activated through the usual UPR induced transcription factors.

## Results

### Activation of the eIF2α-phosphorylation dependent regulatory pathway during a heat shock

**Figure 1:**
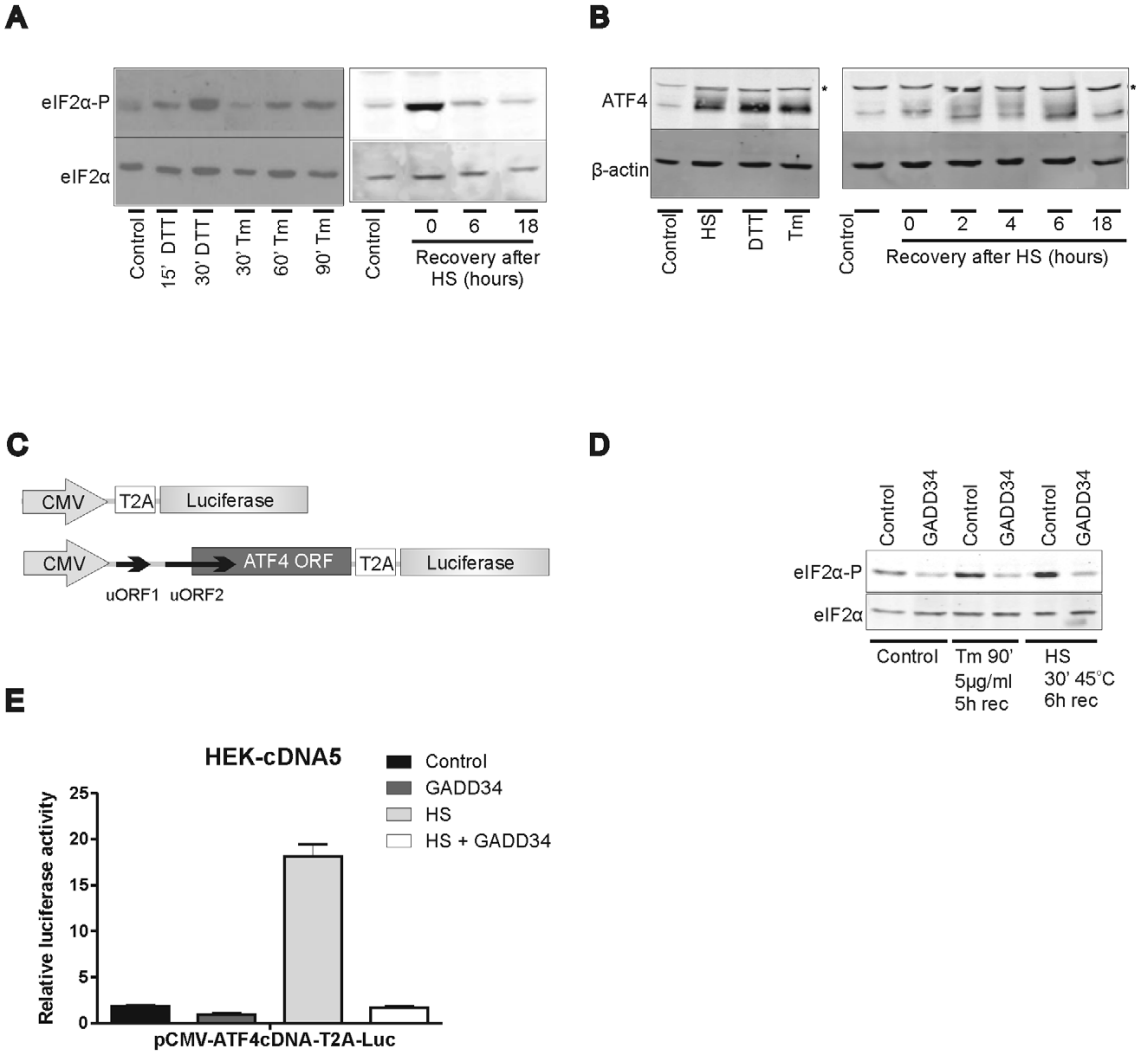
eIF2α-P dependent translation of the ATF4 ORF upon heat stress. (A, B) eIF2α-P and ATF4 levels in ER or heat stressed cells. HEK-cDNA5 cells were treated with 10 mM DTT or 5 µg/ml tunicamycin for the indicated time to induce ER stress. Alternatively cells were exposed to a heat shock of 30′ at 45°C (HS) or left at 37°C (37°C). When heat shocked, cells were allowed to recover for the indicated time before harvesting. Cell lysates were subjected to SDS-PAGE and levels of phosphorylated eIF2α (eIF2α-P) were determined by western blotting with eIF2α as a loading control (panel A) or levels of ATF4 were determined by western blotting with β-actin as a loading control (panel B). The asterisk indicates a non specific band. (C) Schematic representation of the luciferase reporter constructs containing the ATF4 ORF. Translation of the luciferase code is dependent upon translation of the preceding ATF4 open reading frame. (D) Expression of C-term GADD34 decreases the level of eIF2α-P. HEK-cDNA5 cells were transfected with GADD34 or with an empty vector. Cell lysates of unstressed HEK-cDNA5 cells or HEK-cDNA5 cells exposed to tunicamycin or HS were subjected to SDS-PAGE and levels of phosphorylated eIF2α (eIF2α-P) were determined by western blotting. eIF2α was used as a loading control. (E) Translation of the ATF4 ORF in heat shocked cells is eIF2α-P dependent. HEK-cDNA5 cells were transfected with a mixture (4∶1∶5) of the indicated luciferase reporter, a βactin-βgal reporter, and the expression construct for C-term GADD34 or an empty vector. At 48 h after transfection, cells were exposed to a heat shock of 30′ at 45°C (HS) or left at 37°C (Control). When heat shocked, cells were allowed to recover for 7 hours and harvested. Harvested cells were assayed for reporter gene activities. The results are the average of three independent transfections (standard deviations are indicated by error bars).

During both ER stress and heat stress eIF2α is phosphorylated (Fig. 1A), where heat stress induced phosphorylation of eIF2α is most likely mediated by PKR and HRI, while ER stress activates PERK. When eIF2α is phosphorylated. ATF4 mRNA is selectively translated and a similar increase in ATF4 levels in cells recovering from heat shock and in DTT or tunicamycin treated cells was seen (Fig. 1B; the multiple ATF4 bands most likely represent phosphorylated forms [36]). eIF2α phosphorylation decayed rapidly in cells recovering from heat shock (Fig. 1A) and to show that the increase in ATF4 is indeed due to eIF2α-P dependent *de novo* translation, we designed reporter constructs in which translation of the luciferase code is dependent upon translation of the preceding ATF4 ORF by linking that ORF to the luciferase code with the T2A viral frameshift region. Upon translation of the T2A sequence, the ribosome frameshifts, releasing the protein, but continues translation of the downstream ORF [37], [38], in our constructs the luciferase coding region (see fig. 1C). As expected, placing the ATF4 cDNA sequence, including the upstream ORFs, before the T2A-luciferase ORF was strongly inhibitory: the luciferase yield from the pCMV-ATF4 constructs was in unstressed cells about 5% of that obtained from the control, pCMV-T2A-luc (data not shown). Dephosphorylating the little eIF2α-P present in unstressed cells by expressing the C-terminal domain of GADD34 (C-term GADD34), a constitutively active mutant of the regulatory subunit of eIF2α dephosphorylase, caused a further decrease of the luciferase yield (the efficacy of exogenous expression of the C-term GADD34 in dephosphorylation of eIF2α-P is shown in fig. 1D). Heat stressing the cells resulted in a sharp increase in luciferase yield from the ATF4 constructs, an increase that is completely prevented by exogenous expression of C-term GADD34 (Fig. 1E). These data show that the increase in ATF4 levels in heat stressed cells is due to eIF2α-P dependent translation initiation just as it is in ER stressed cells.

### Activation of the ATF6 dependent regulatory pathway during a heat shock

**Figure 2:**
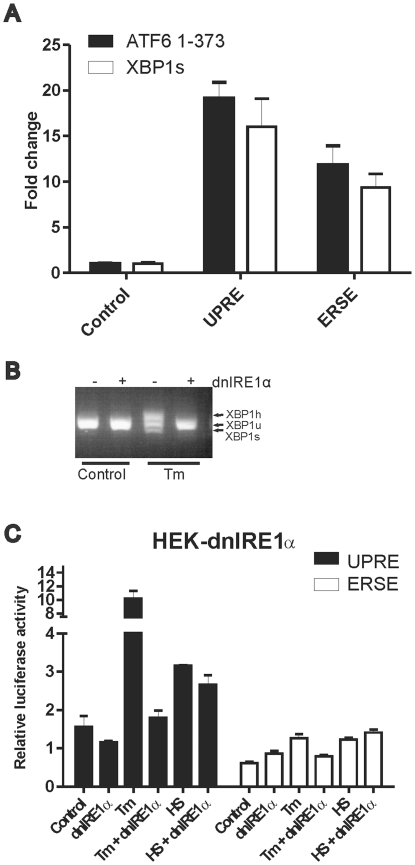
UPRE and ERSE directed reporter gene activity. (A) Effect of exogenous expression of ATF6 or spliced XBP1 on the activity of the UPRE-luciferase and ERSE-luciferase reporter constructs. HEK-cDNA5 cells were transfected with a mixture (4∶1∶5) of the indicated luciferase reporter, a βactin-βgal reporter, and pcDNA3.1-ATF6α (1–373), pcDNA5-XBP1s or an empty vector pcDNA5/FRT/TO. At 48 hours after transfection, cells were harvested and assayed for reporter gene activities. (B) dnIRE1α blocks XBP1 splicing. HEK-dnIRE1α cells were cultured in the absence or presence of doxycycline. Cells were treated with 10 µg/ml tunicamycin for 90′ to induce XBP1 splicing. Total RNA samples were analyzed by RT-PCR. PCR products represent unspliced (XBP1u), spliced (XBP1s) and a hybrid of spliced and unspliced XBP1 PCR products (XBP1h; see also Materials and methods). (C) HEK-cDNA5 and HEK-dnIRE1α cells were transfected with a mixture (9∶1) of the indicated luciferase reporter and a βactin-βgal reporter. At 24 hours after transfection dnIRE1α expression was induced by adding doxycycline. At 48 hours after transfection, cells were exposed to a heat shock of 30′ at 45°C (HS) or left at 37°C (37°C). When heat shocked, cells were allowed to recover for 18 hours and harvested. To induce ER stress, cells were exposed to 2 µg/ml tunicamycin for 24 hours. Harvested cells were assayed for reporter gene activities.

The data presented above show that the ATF4 branch of the UPR is also activated in heat stressed cells. To determine whether a heat stress also activates the ATF6 arm of the UPR we used two reporter genes. One is driven by the UPRE [39]; the other by the ERSE [40]. Both DNA elements are targeted by ATF6 as evidenced by the increased activity upon exogenous expression of the soluble form of ATF6 (ATF6 1–373), but not uniquely, as these elements are also targets of XBP1s (Fig. 2A). To exclude an effect of XBP1s we inactivated this branch of the UPR by expression of a dominant negative mutant of IRE1α. Fig. 2B shows that expression of dnIRE1α inhibited tunicamycin induced XBP1 splicing. The activity of the UPRE reporter increased more than six fold after tunicamycin induced ER stress, while heat shock caused only a twofold induction. DnIRE1α expression strongly inhibited the ER stress induced activity, while the mild activation after heat shock was still observed (Fig. 2C left, black bars). The ERSE reporter was less responsive to stress and an increase of only twofold was seen after heat shock or tunicamycin treatment. In presence of dnIRE1α, tunicamycin no longer induced the reporter, while the heat shock induced activity remained. (Fig. 2C right, white bars) These results suggest that the increased activity of both the UPRE and the ERSE driven reporter genes in tunicamycin treated cells, but not in heat shocked cells, is dependent upon XBP1s. Only the UPRE driven reporter gene shows a slight activation in tunicamycin treated dnIREα cells, potentially due to ATF6. If so, ATF6 could also be responsible for the XBP1s independent activity seen in heat shocked cells. The activity of the ERSE driven reporter gene is completely dependent upon XBP1 in tunicamycin cells, no sign of a possible ATF6 contribution is seen. Hence we cannot conclude that the heat shock induced activity of this reporter is due to ATF6; it may well be due to other transcription factors.

### Heat shock induces XBP1 mRNA splicing

**Figure 3:**
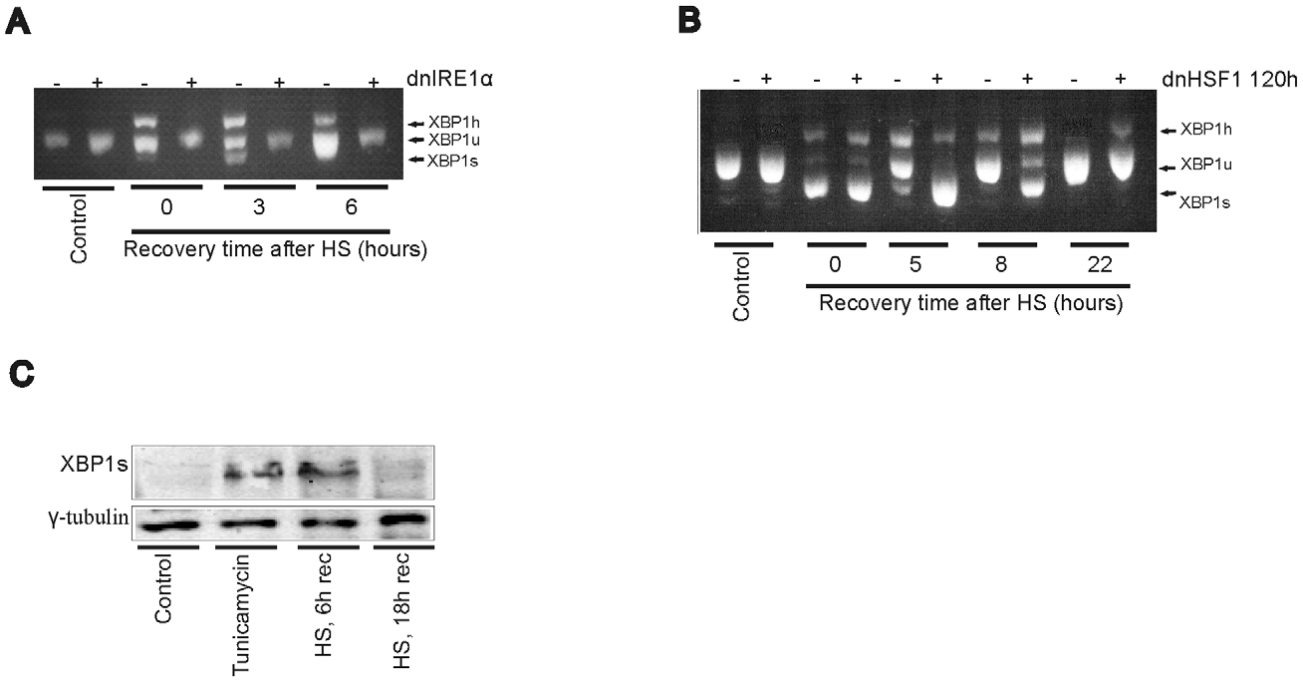
XBP1 splicing in heat shocked cells. (A) HEK-dnIRE1α cells were cultured in the absence or presence of doxycycline. Cells were exposed to a heat shock of 30′ at 45°C (HS) or left at 37°C (no stress). When heat shocked, cells were allowed to recover for the indicated periods at 37°C. Total RNA samples were analyzed by RT-PCR. (B) HEK-dnHSF1 cells were cultured in the absence or presence of doxycycline for the indicated time. Cells were exposed to a heat shock (30′, 45°C), harvested at the indicated time point after heat shock, and subjected RT-PCR analysis to investigate the effect of HEK-dnHSF1 on XBP1 splicing after heat stress. (C) XBP1s levels in heat stressed cells. HEK-cDNA5 cells were exposed to a heat shock of 30′ at 45°C or left at 37°C. When heat shocked, cells were allowed to recover for the indicated time before harvesting. To induce ER stress, cells were treated with 5 µg/ml tunicamycin for 90 minutes. After 5 hours recovery cells were harvested. Cell lysates were subjected to SDS-PAGE and levels of XBP1s were determined by western blotting with γ-tubulin as a loading control.

The XBP1 branch of the UPR has been reported to be activated in heat shocked cells [34], and we therefore expected to see an inhibition of the UPRE driven reporter gene in heat shocked dnIRE1α cells. However, we saw little effect (see Fig. 2C) and thus looked to see if XBP1 mRNA is spliced in heat shocked cells under our experimental conditions. Directly after heat shock or after 3 hrs of recovery, XBP1 mRNA was indeed completely spliced (Fig. 3A). After about 8 hrs unspliced XBP1 mRNA was again detected (Fig. 3B). Figure 3A shows that heat-induced XBP1 splicing was strongly inhibited by the expression of dnIRE1α, indicating that heat shock induced XBP1 splicing is, as expected, IRE1α dependent. Recovery from XBP1 splicing is dependent on a healthy heat shock system. In heat stressed MEF HSF1 −/− cells XBP1 splicing was prolonged [34]. In agreement, we found that expression of a dominant negative HSF1 mutant [41] also delays the reappearance of unspliced XBP1 after heat stress (Fig. 3B). XBP1 splicing in cells recovering from heat stress is transient and XBP1 protein can no longer be detected in cells that have recovered for 18 hrs from a heat shock, although it is present 6 hrs after heat shock (Fig. 3C).

### The DNAJB9 promoter becomes activated upon heat stress, independent of XBP1s

**Figure 4:**
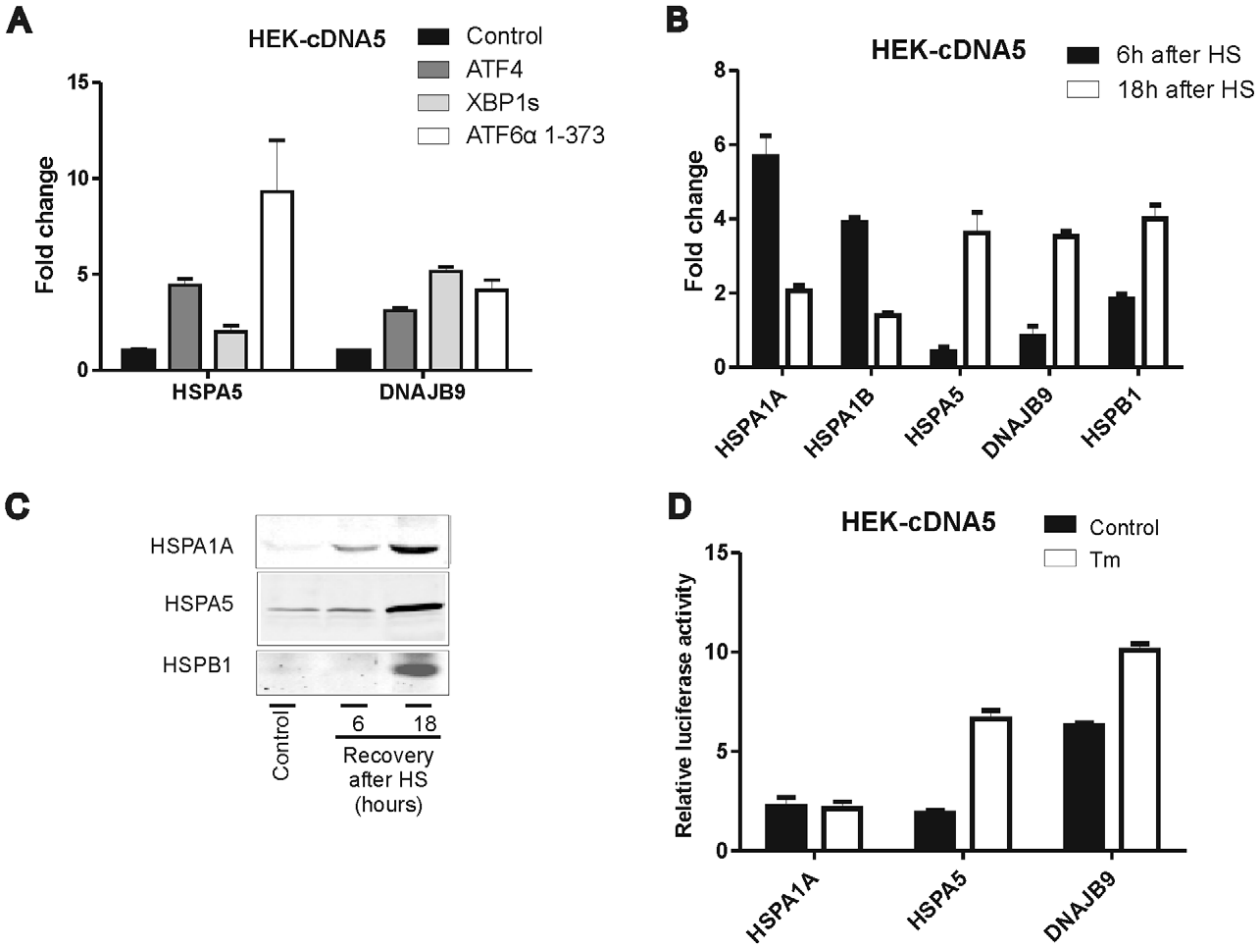
Stress response of various promoters. (A) ATF4, ATF6 and XBP1s activate the HSPA5 and DNAJB9 promoters. HEK-cDNA5 cells were transfected with a mixture (4∶1∶5) of the indicated luciferase reporter, a β-actin-βgal reporter, and pcDNA5-XBP1s, pcDNA5-ATF4ORF, pcDNA3.1-ATF6α (1–373) or the empty vector pcDNA5/FRT/TO. At 24 hours after transfection doxycyclin was added to induce expression of XBP1s or ATF4. At 48 hours after transfection, cells were harvested and assayed for reporter gene activities. For the levels of exogenously expressed XBP1 and ATF4, see Fig. S1. (B) Heat shock response of various promoters. HEK-cDNA5 cells were transfected with the indicated promoter reporter construct and a β-actin-β-galactosidase reporter (9∶1 ratio). At 48 h after transfection, cells were exposed to a heat shock of 30′ at 45°C or left at 37°C. When heat shocked, cells were allowed to recover for 6 hours or 18 hours, and assayed for reporter gene activities. The luciferase activity of the promoter constructs is relative to the activity of pGL3 control. (C) HEK-cDNA5 cells were exposed to a heat shock of 30′ at 45°C or left at 37°C. When heat shocked, cells were allowed to recover for the indicated time before harvesting. Cell lysates were subjected to SDS-PAGE and levels of HSPA1A, HSPA5 and HSPB1 were determined by western blotting. (D) ER stress activation of various promoters. HEK-cDNA5 cells were transfected with the indicated promoter reporter construct and a β-actin-β-galactosidase reporter (9∶1 ratio). At 48 h after transfection, cells were exposed to 2 µg/ml tunicamycin for 18 hours, and assayed for reporter gene activities. The luciferase activity of the promoter constructs is relative to the activity of pGL3 control.

The data presented above show that the UPR is induced by heat shocking cells but leave some doubt as to whether the typical UPR transcriptional response is also seen: an UPRE driven reporter gene was only inhibited by dnIRE1α in tunicamycin treated cells, not in heat shocked cells (Fig. 2C). We thus tested the heat shock inducibility of two canonical UPR promoters, the human DNAJB9 (ERdj4) promoter and HSPA5 (BiP) promoter. The DNAJB9 promoter is a target of XBP1s [42], [43] and ATF4 [44]; the HSPA5 promoter is activated by ATF6 via an ERSE [45] and also by ATF4 [46]. The ATF4, ATF6 and XBP1s sensitivity of our HSPA5 and DNAJB9 promoter constructs was tested by assaying their response to exogenous expression of either ATF4, ATF6 or XBP1s. The HSPA5 promoter was most sensitive to exogenous ATF6 expression (Fig. 4A, white bars) while the DNAJB9 promoter responded best to exogenous XBP1s expression (Fig. 4A, light gray bars; see Fig. S1 for expression levels of exogenous XBP1s and ATF4 protein). In cells recovering from a heat shock for 6 hrs, luciferase constructs driven by either the DNAJB9 or the HSPA5 promoter were not active, while human HSPA1A (Hsp70) or HSPA1B promoter driven luciferase genes were fully active. However, a HSPB1 promoter driven luciferase gene was also inactive, even though the HSPB1 promoter is a canonical heat shock promoter (Fig. 4B). When the activity of the same constructs is assayed in cells that were allowed to recover from a heat shock overnight (18 hrs), the activity of the HSPA1 promoter driven constructs was already decaying (relative to pGL3 control), but that of the HSPB1, DNAJB9 and HSPA5 promoter constructs was higher. Apparently these three promoters are delayed responders to a heat shock. The delayed activity of at least the HSPB1 and HSPA5 promoter constructs reflects that of the endogenous genes: the protein products were only detectable in cells after overnight recovery from the heat shock (Fig. 4C). As expected, the activity of the HSPA5 and DNAJB9 promoters increased upon tunicamycin treatment of the cells, while that of the HSPA1A promoter did not (Fig. 4D).

**Figure 5:**
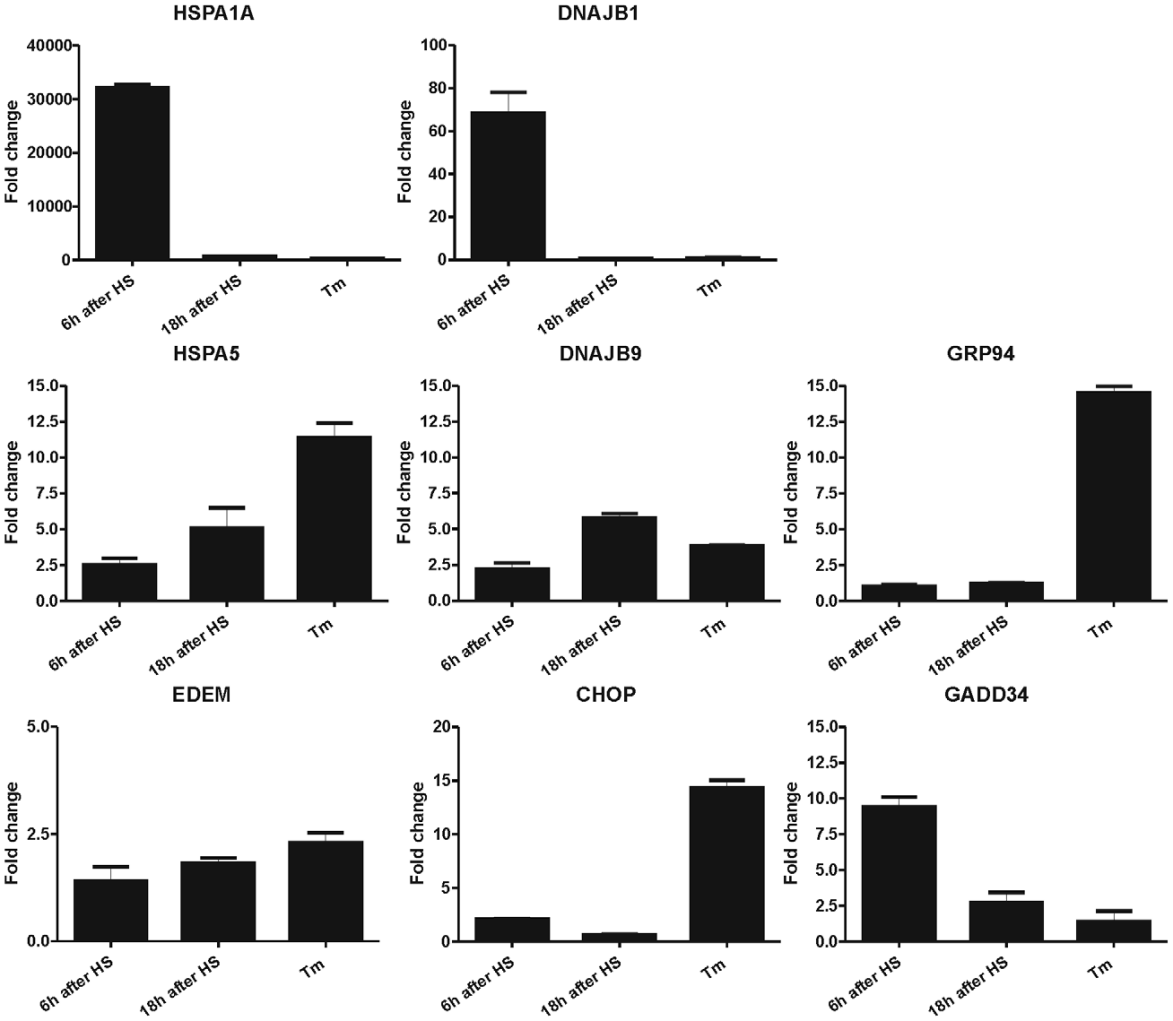
Relative changes in transcript levels of ER responsive genes in heat shocked and tunicamycin treated cells. HEK-cDNA5 cells were treated with 2 µg/ml tunicamycin for 24 hours to induce ER stress. Alternatively cells were exposed to a heat shock of 30′ at 45°C (HS) or left at 37°C (37°C). When heat shocked, cells were allowed to recover for the indicated time before harvesting. Total RNA was isolated and transcript levels were measured by QPCR. Fold induction of mRNA levels is plotted relative to GAPDH mRNA levels.

The increased activity of the HSPA5 and DNAJB9 promoters in heat shocked cells raised the question whether other ER stress responsive genes are also activated. We thus looked at the changes in transcript levels of six ER stress responsive genes (HSPA5, DNAJB9, GRP94, EDEM, CHOP and GADD34) in heat shocked or tunicamycin treated cells and compared this with the changes in HSPA1A and DNAJB1 mRNA levels (Fig. 5). The transcript levels of the ER responsive genes all increased upon tunicamycin treatment and, with the exception of DNAJB9 and GADD34, were higher than in heat shocked cells. The HSPA1A and DNAJB1 mRNA levels did not increase in tunicamycin treated cells. A (modest) increase in the transcript level of all genes tested was seen in cells 6 hours after heat shock. Transcript levels were generally higher in cells 18 hrs after heat shock, with the exception of the two canonical ATF4 target genes (CHOP and GADD34) and the two canonical heat shock genes (HSPA1A and DNAJB1). These data show that these six ER responsive genes are active in heat shocked cells; generally, as also indicated by the activity of the promoter constructs, as a delayed response. The relative level of the transcripts in heat shocked cells is, however, different from that in tunicamycin treated cells (Fig. 5).

**Figure 6:**
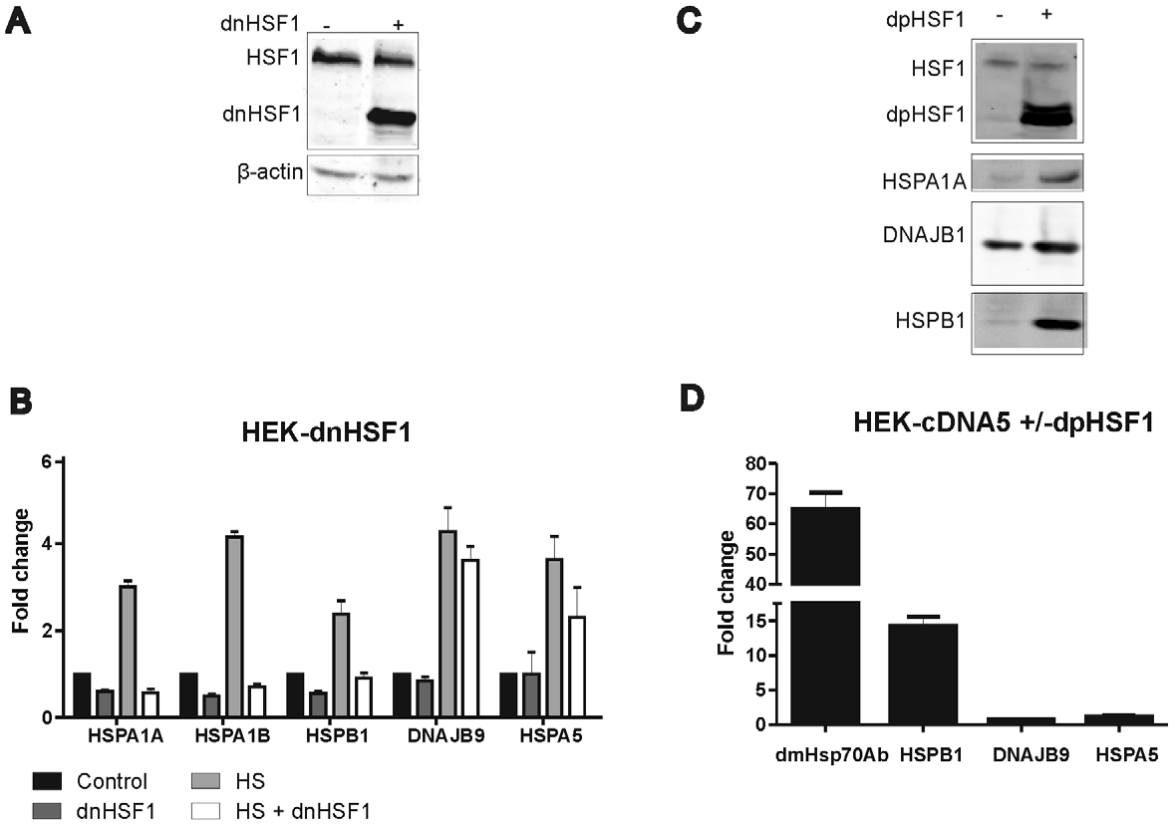
HSF1 dependency of various promoters. (A) Expression of dnHSF1. HEK-dnHSF1 were cultured in the absence or presence of doxycycline to induce expression of dnHSF1. After harvesting the cells were subjected to SDS-PAGE and levels of endogeneous HSF1 and dnHSF1 were determined by western blotting with β-actin as a loading control. (B) The effects of dnHSF1 on basal and heat shock induced activity of various promoters. Cells were transfected with a mixture of the indicated luciferase reporter and a β -actin-β-galactosidase reporter (9∶1 ratio). At 24 hours after transfection doxycyclin was added to induce expression of dnHSF1. At 48 hours after transfection, cells were exposed to a heat shock of 30′ at 45°C or left at 37°C. When heat shocked, cells were allowed to recover for the indicated periods at 37°C. Harvested cells were assayed for reporter gene activities. (C) Expression of dpHSF1 leads to increased HSP levels. HEK-cDNA5 cells were tranfected with pcDNA5-dpHSF1. At 24 hours after transfection cells were cultured in the absence or presence of doxycycline to induce expression of dpHSF1. At 48 hours after transfection cells were harvested and subjected to SDS-PAGE and levels of endogenous HSF1, dpHSF1, HSPA1A, DNAJB1 and HSPB1 were determined by western blotting. (D) The effect of dpHSF1 on the activity of various promoters. HEK-cDNA5 cells were transfected with a mixture (4∶1∶5) of the indicated luciferase reporter, a βactin-βgal reporter, and pcDNA5-dpHSF1. At 24 hours after transfection doxycycline was added to induce expression of dpHSF1. At 48 hours after transfection, cells were harvested and assayed for reporter gene activities.

To determine whether the heat shock induced activity of the HSPA5 and DNAJB9 promoters (see Fig. 4B) is a result of the activity of the transcription factor HSF1, we tested promoter activity in HEK-dnHSF1 cells. As shown in figure 6B, the expression of dnHSF1 effectively blocks the heat shock induced activity of the DmHsp70Ab, the HSPA1A, the HSPA1B, and the HSPB1 promoter constructs, but not that of the DNAJB9 promoter construct, while the HSPA5 promoter construct is only slightly inhibited (note that the levels of HSF1 and dnHSF1 remain constant in cells recovering from heat shock; Fig. S2). To confirm that the DNAJB9 and the HSPA5 promoter constructs do not respond to HSF1, we also tested the effect of a dominant positive HSF1 mutant. Expression of this mutant strongly upregulated the endogenous HSPA1A, DNAJB1 and HSPB1 protein levels, indicating an activated heat shock response (Fig. 6C). Expression of dpHSF1 also resulted in increased activity of the DmHsp70Ab and the HSPB1 promoter constructs in the absence of stress, yet no effect was seen for the DNAJB9 or HSPA5 promoter constructs (Fig. 6D). We conclude that the DNAJB9 and HSPA5 promoters are not a target of HSF1.

**Figure 7:**
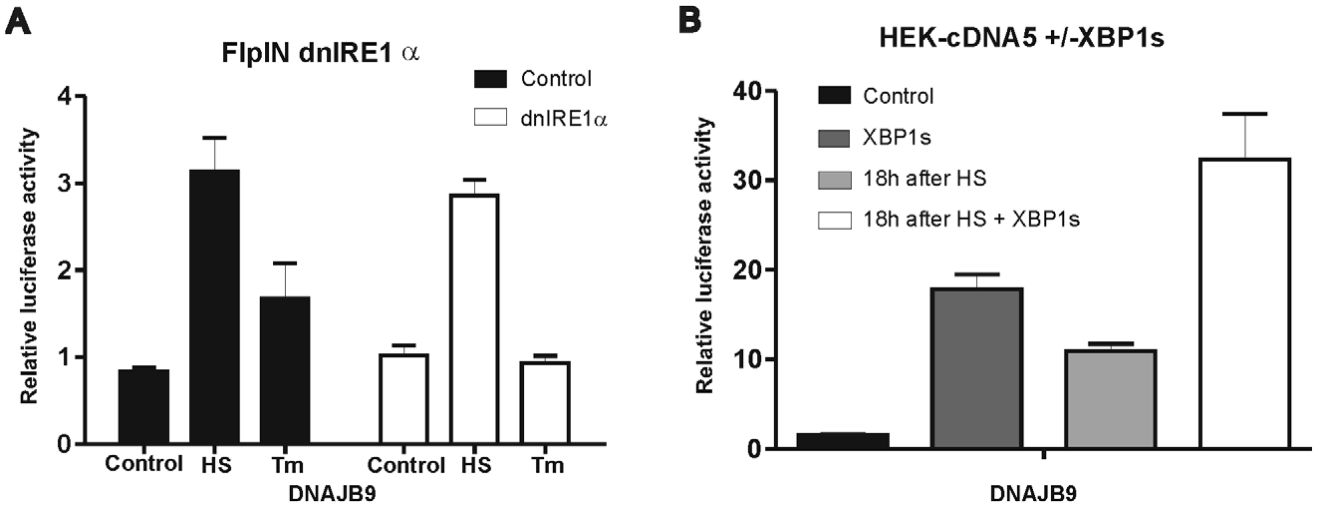
DNAJB9 promoter activity is not regulated by XBP1s in heat shocked cells. (A) Activity of the DNAJB9 promoter in HEK-dnIRE1α cells. HEK-dnIRE1α cells were transfected with a mixture of the indicated luciferase reporter and a β -actin-β-galactosidase reporter (9∶1 ratio). At 24 hours after transfection doxycyclin was added to induce expression of dnIRE1α. At 48 hours after transfection, cells were exposed to a heat shock of 30′ at 45°C or left at 37°C. When heat shocked, cells were allowed to recover for 18 hours and harvested. Alternatively, cells were exposed to 2 µg/ml Tunicamycin for 18 hours. Harvested cells were assayed for reporter gene activities. (B) The DNAJB9 promoter can be activated by exogenous XBP1s. HEK-cDNA5 cells were transfected with a mixture (4∶1∶5) of the indicated luciferase reporter, a βactin-βgal reporter, and pcDNA5-XBP1s or the empty vector pcDNA5/FRT/TO. At 24 hours after transfection doxycyclin was added to induce expression of XBP1s. At 48 hours after transfection, cells were exposed to a heat shock of 30′ at 45°C or left at 37°C. When heat shocked, cells were allowed to recover for 18 hours, harvested and assayed for reporter gene activities.

**Figure 8:**
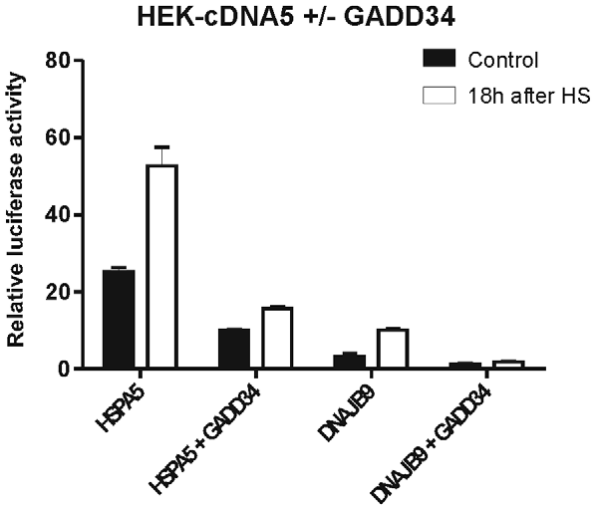
eIF2α-P dependent DNAJB9 and HSPA5 promoter activity. Activity of DNAJB9 and HSPA5 promoter in heat shocked cells is eIF2α-P dependent. HEK-cDNA5 cells were transfected with a mixture (4∶1∶5) of the indicated luciferase reporter, a βactin-βgal reporter, and the expression construct for GADD34 or an empty vector. At 48 hours after transfection, cells were exposed to a heat shock of 30′ at 45°C (HS) or left at 37°C (37°C). When heat shocked, cells were allowed to recover for 18 hours and harvested. Harvested cells were assayed for reporter gene activities.

The activation of canonical UPR promoters in cells recovering from a heat shock does suggest that heat shocked cells mount the UPR response. We thus tested whether the DNAJB9 promoter construct is activated by a heat shock when the XBP1 branch of the UPR is blocked. As shown in figure 7A, in tunicamycin treated cells, activation of the DNAJB9 promoter construct is fully dependent upon XBP1s, as the response is absent in dnIRE1α cells. However, in heat shocked dnIRE1α cells, the DNAJB9 promoter construct is fully active. Hence endogenous XBP1s is not involved in the heat shock activation of the DNAJB9 promoter. XBP1s is not inactivated in heat shocked cells, as bypassing IRE1α by exogenous expression of XBP1s did increase the activity of the DNAJB9 promoter (Fig. 7B). One possibility is that the DNAJB9 promoter construct is activated by ATF4, which is also upregulated in heat shocked cells (Fig. 1). ATF4 synthesis requires eIF2α phosphorylation and we thus checked whether activation of the DNAJB9 construct was dependent upon eIF2α phosphorylation. As control we used the HSPA5 promoter construct, known to be induced by eIF2α phosphorylation [46]. As shown in figure 8, the activity of these two promoter constructs both in unstressed and in heat stressed cells was indeed blocked when eIF2α is dephosphorylated by exogenous expression of the C-terminal domain of GADD34, showing that the transcriptional activation is the consequence of stress induced differential translation.

**Figure 9:**
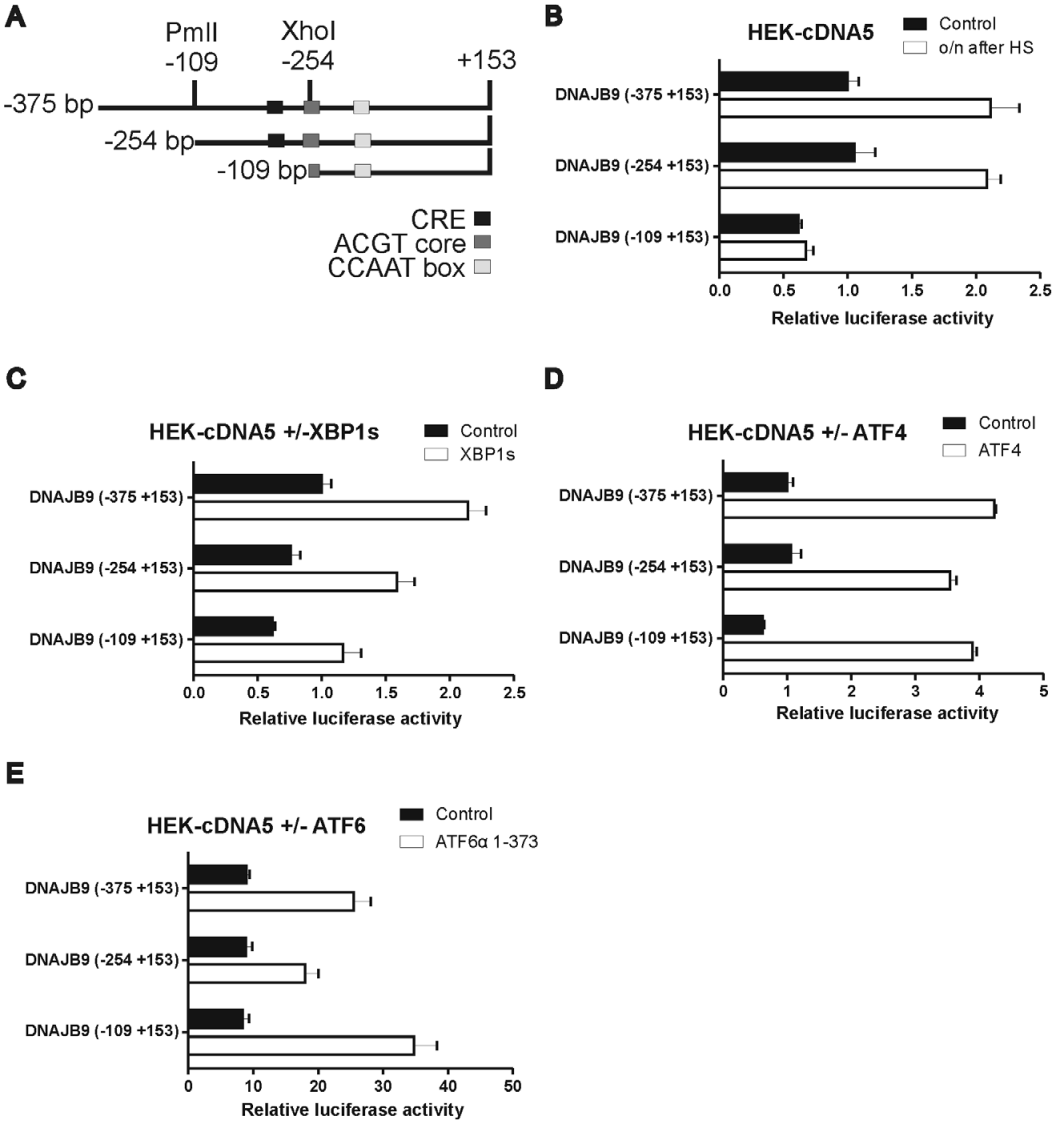
DNAJB9 promoter deletion constructs. (A) Schematic representation of the DNAJB9 promoter region. (B) Heat shock inducibility of DNAJB9 deletion constructs. HEK-pcDNA5 cells were transfected with a mixture of the indicated luciferase reporter and a βactin-β-galactosidase reporter (9∶1 ratio). At 48 hours after transfection, cells were exposed to a heat shock of 30′ at 45°C or left at 37°C. When heat shocked, cells were allowed to recover for 18 hours, harvested and assayed for reporter gene activities. (C,D,E) Effect of exogenous XBP1s, ATF4 or ATF6 expression on the activity of promoter deletion constructs. HEK-cDNA5 cells were transfected with a mixture (4∶1∶5) of the indicated luciferase reporter, a βactin-βgal reporter, and an expression construct as indicated or the empty vector pcDNA5/FRT/TO. At 24 hours after transfection doxcycyclin was added to induce XBP1s (C) or ATF4 expression (D). At 48 hours after transfection, cells were harvested and assayed for reporter gene activities.

If the transcription factor involved in the heat shock activation of the DNAJB9 promoter is ATF4, then the ATF4 responsive element in that promoter should also be the element mediating the heat shock inducibility of the DNAJB9 promoter. The DNAJB9 promoter contains a CRE-like element around −140 and a CCAAT box around −65. ATF4 preferentially binds a CRE, but was also shown to bind CCAAT box although less efficiently [47]. Deleting the promoter to −109 resulted in loss of the heat shock inducibility but the truncated promoter could still be activated by ATF4 and XBP1s (Fig. 8). Hence the DNA region required for heat shock inducibility and that required for activation by ATF4 do not co-localize and we therefore conclude that the heat shock induction of the DNAJB9 promoter is not mediated by ATF4 (see Fig. 9). The heat insensitive DNAJB9 promoter construct was also still activated by exogenous expression of ATF6, also excluding ATF6 as the transcription factor responsible for DNAJB9 promoter activation after heat shock.

## Discussion

In agreement with a previous study [34], we found that a heat shock elicits the typical ER stress markers: phosphorylation of eIF2α and splicing of XBP1 mRNA. In addition, we show that ATF4 is synthesized, that XBP1s is made, that consensus ERSE and UPRE elements direct transcriptional activation in heat shocked cells and that canonical UPR promoters, HSPA5 and DNAJB9, are activated in heat shocked cells as also seen previously in microarray studies [35]. The response resembles a typical UPR, albeit with some differences in the relative mRNA levels. However, when we tried to identify the transcription factors involved in the transcriptional activation of UPR promoters in heat shocked cells, we found that those factors differ from the traditional ones responsible for the UPR (ATF4, XBP1 and ATF6). XBP1s plays a major role in the transcriptional activation directed by the ERSE or UPRE elements in tunicamycin treated cells, but is not involved in the transcriptional activation directed by these elements after a heat shock as the heat shock response is not blocked in dnIRE1α cells. Similarly, when the response of the DNAJB9 promoter is further dissected, then it is clear that the activation of this promoter during heat shock is distinct from the activation during the UPR: deleting the promoter to −109 does not affect the activation by ATF4 or ATF6, has some effect on the activation by XBP1s but the heat shock induction is lost. Hence neither ATF4, ATF6 nor XBP1s plays a role in the heat shock activation of this promoter. Our experiments also exclude that the DNAJB9 promoter is a target of HSF1. As blocking eIF2α phosphorylation also blocks the heat shock response of the DNAJB9 (and of the HSPA5) promoter, the activity of the transcription factor responsible is somehow translationally controlled. We have tested ATF5 and CHOP (a target of ATF4) but neither activated either the DNAJB9 or the HSPA5 promoter (data not shown).

eIF2α phosphorylation is a common response to different stressors, not only cytoplasmic or ER proteotoxic stress, but also lack of amino acids [48]. ATF4 synthesis is thus also common to various types of stress and the pattern of transcriptional activation by ATF4 needs to be tailored to fit the type of stress. The targets of ATF4 are thus also determined by stress specific auxiliary factors [49]. Therefore, it is perhaps not surprising that ATF4 does not transactivate all its usual UPR targets during the heat shock response. XBP1s synthesis however is uniquely controlled by IRE1α dependent splicing and we expected XBP1s to be active irrespective of other stress responses. XBP1s transactivation activity itself is not blocked in heat shocked cells, as exogenous XBP1s does activate the DNAJB9 promoter construct in heat shocked cells (Fig. 7). Endogenous XBP1s activity in heat shocked cells is predicted to be transient. We find only a minor fraction of spliced XBP1 mRNA in cells recovering for 5 hrs from a heat shock and we do not detect XBP1s in cells that have recovered for 18 hrs. It is possible that the activity of endogenous XBP1s is masked by that of another transcription factor targeting the same DNAJB9 promoter. Alternatively, endogenous XBP1s may be inactivated by acetylation [50] or sumoylation [51] during the first few hours after heat shock. Exogenous XBP1s, which continues to be expressed, might escape from such inhibition at later times.

At least in yeast the UPR becomes activated during heat stress, in addition to the heat shock response [52], [53]. Here, the heat shock response and the UPR cooperate and the heat shock response can rescue a defective UPR [54]. Our data suggest that after a heat shock mammalian cells first devote their resources to increasing the cytoplasmic chaperoning capacity – and only later switch to augmenting the ER chaperones. Accumulation of HSPA1A is detected within 6 hrs after heat shock, while HSPA5 levels increase later (Fig.4). At the time that the DNAJB9 and the HSPA5 promoters become active, the heat shocked induced UPR (as indicated by XBP1 splicing) has already decayed. Perhaps that is why another set of transcription factors needed to be recruited. This set of transcription factors could also be active in ER-stressed cells, and thus be part of the traditional UPR response, but have gone undetected because of the overlapping ATF4, ATF6 and XBP1 activity.

## Materials and Methods

### Cell culture

Flp-In T-REx-HEK293 cells (Invitrogen) were manipulated according to the manufacturer's instructions to generate the stable cell lines HEK-dnIRE1α, HEK-dnHSF1 and HEK-cDNA5, which carry a single copy of the tetracycline-inducible plasmids pcDNA5- dnIRE1α, pcDNA5-dnHSF1, and pcDNA5-FRT/TO, respectively. The cells were cultured at 37°C in the presence of humidified 5% CO2 in high glucose DMEM medium supplemented with 10% fetal calf serum and 100 U/ml penicillin and 100 µg/ml streptomycin. Blasticidin (1.65 µg/ml; Invitrogen) and 100 µg/ml hygromycin were also added to the culture medium during maintenance of the cell lines, but were omitted during experiments.

### Plasmid Construction

The C-terminal truncation mutant of dnHSF1 containing codons 1–379 from HSF1 was previously described [41]. The truncated dnIRE1α lacks the sequence of kinase and ribonuclease domains [55]. pGEM-T-hIRE1αDelC-29 was made by PCR amplifying the 1.75-kb IRE1α cDNA from HEK293 cDNA using the IRE1up and IRE1-delClow primers and cloning the PCR fragment into the pGEM-T vector. pcDNA5-FRT-TO-IRE1αDelC was made by cloning the 1.75-kb HindIII-XhoI fragment of pGEM-T-hIRE1αDelC-29 into the HindIII and XhoI sites of pcDNA5/FRT/TO. pcDNA5-dpHSF1 was made by digesting pcDNA5/FRT/TO-dnHSF1 with BamHI(bl) and XhoI(bl) resulting in HSF1 AA1-201, then the SmaI-BglII(bl) fragment from pOTB-HSF1 (C-terminal fragment of HSF1 AA316–529) was inserted into HSF1 1–201, generating pcDNA5/FRT/TO-HSF1Δ202–315 (dpHSF1).

The reporter constructs were made in the pGL3 basic vector (Promega). The *Drosophila melanogaster* Hsp70 (Hsp70Ab), HSP70A1A (−313, +196), HSPB1 (−685, +36) and HSPA5 (−2742, +202) promoter constructs were described previously [32], [41]. The HSPA1B (−573, +13), and the DNAJB9 (−375, +153) promoter clones were constructed by PCR on DNA isolated from human lymphocytes cells using the primers listed in table 1. PCR fragments were cloned into pGEM-T-Easy vector (Promega) and, after sequencing; the promoter sequence was cloned into the pGL3 basic vector. DNAJB9 promoter deletion constructs were generated by digesting pGL3- DNAJB9 (−376, +153) with SacI and XhoI (−254, +153) or PmlI (−109, +153). Blunt ends were generated and ligated.

**Table 1:** Oligonucleotides.

Oligo Name	Oligo Sequence (5′ ->3′)
IRE1 up	agctaagcttaccatgccggcccggcggct
IRE1delC low	agctctcgagtcatccatggcccaggacatccttg
HSPA1B promoter up	agctctcgagaactatattgcattatctctttcct
HSPA1B promoter low	agctagatctggccgttttccggac
DNAJB9 promoter up	aacgcgtcttacaacaaatcgctgcgcaatag
DNAJB9 promoter low	accatggtggcgctggcgaccctgacgatc
XBP1s expression up	agctaagcttaccatggtggtggtggcagccgc
XBP1s expression low	agctctcgagttagacactaatcagctggggaa
XBP1 PCR up	ctggaacagcaagtggtaga
XBP1 PCR low	tctacccagaaggacccagt
hATF4 up	agctaagctttttctactttgcccgcccacag
hATF4 low	agctgaattcggggacccttttcttccccct
pcDNA5-ATF4ORF up	agctaagcttctcacggcattcagcagc
pcDNA5-ATF4ORF low	agctgaattccaccacactggactag
ERSE up	ctagccgaccaatgatggtcgaccacgcgtgg
ERSE low	gatcccacgcgtggtcgaccatcattggtcgg
UPRE RE up	ctagcacaggtgctgacgtggcattca
UPRE RE low	gatctgaatgccacgtcagcacctgtg
EDEM QPCR up	aaagattccaccgtccaagtc
EDEM QPCR low	gtatcattgctccggaggtt
HSPA5 QPCR up	ggccgcacgtggaatgac
HSPA5 QPCR low	tccaatatcaacttgaatgtatgg
HSPC4 (GRP94) QPCR up	ctgggactgggaacttatgaatg
HSPC4 (GRP94) QPCR low	tccatattcgtcaaacagacca
CHOP QPCR up	accaagggagaaccaggaaacg
CHOP QPCR low	tcaccattcggtcaatcagagc
GADD34 QPCR up	cctctacttctgccttgtctccag
GADD34 QPCR low	ttttcctccttctcctcggacg
DNAJB9 QPCR up	tggtggttccagtagacaaagg
DNAJB9 QPCR low	cttcgttgagtgacagtcctgc
DNAJB1 QPCR up	ttccccagacatcaagaacc
DNAJB1 QPCR low	accctctcatggtccacaac
HSPA1A QPCR up	ccgagaaggacgagtttgag
HSPA1A QPCR low	acaaaaacagcaatcttggaaagg
GAPDH up	ttccccatggtgtctgagc
GAPDH low	atcttcttttgcgtcgccag

The XBP1s expression construct was generated by PCR on cDNA manufactured from total RNA isolated from Flp-In T-REx 293 cells which were exposed to tunicamycin stress. The PCR product was cloned into the pcDNA5/FRT/TO HindIII-XhoI sites.

pcDNA5/FRT/TO-ATF4-ORF was made by amplifying the 1200-bp ATF4 ORF fragment from pcDNA5/FRT/TO-ATF4cDNA. The PCR fragment was treated with HindIII/EcoRI(bl) and cloned into the HindII and BamH1(bl) sites sites of pcDNA5/FRT/TO. pcDNA3.1-ATF6α (1–373) was kindly provided by Prof. Dr. Kazutoshi Mori [56].

pCMV-ATF4cDNA-T2A-luciferase was made by cloning the 1333 fragment of the ATF4cDNA PCR product (via T-vector) into the HindIII and EcoRI sites of pCMC-T2A-Luc [57]. The constitutively active GADD34 construct has been previously described [58]. pGL3-promoter-UPRE and pGL3-promoter-ERSE were made by annealing the corresponding primers (see Table 1) and cloning the double-stranded oligo into the NheI and BglII sites of pGL3-promoter.

### Reporter assays

At 24 h before transfection, 0.4×105 HEK-cDNA5, HEK-dnIRE1α or HEK-dnHSF1 cells were plated per well in a 24-well plate. Transient transfections were performed using FuGENE-6 (Roche) according to the manufacturer's instructions. 200 ng plasmid was transfected, 20 ng βactin-β-galactosidase was used as a transfection efficiency control. At 24 hours after transfection doxycyclin was added. At 48 hours after transfection cells were harvested or exposed to a stressor. Cells were harvested and lysed in 200 µl reporter lysis mix (25 mM Bicine, 0.05% Tween 20, 0.05% Tween 80) for 10 min. For the β-galactosidase assay, 20 µl cell lysate was mixed with 100 µl Galacton solution (100 mM Na-phosphate pH 8.2, 10 mM MgCl2, 1% Galacton-Plus; Tropix). After 30 min incubation at room temperature, 150 µl accelerator II (Tropix) was added and luminescence was measured with the Lumat LB 9507 tube luminometer (Berthold). For the luciferase assay, 20 µl cell lysate was mixed with 50 µl luciferin solution (Promega) and luminescence was measured with the Lumat luminometer. All reporter gene assays were performed in triplicate. Relative activities of luciferase reporter genes were determined by dividing luciferase values by the corresponding β-galactosidase values to correct for varying transfection efficiencies.

### Total RNA isolation & RT-PCR

RNA isolation, DNase treatment of the RNA and the reverse transcriptase reaction were performed as described [32]. Cells were harvested after the treatments and at the times indicated, washed twice with ice-cold PBS and after washing the cells, RNA was isolated with TRIzol (Invitrogen) according to manufacturer's recommendation. For QPCR analysis, cDNA was 10-fold diluted. Quantitative real-time PCR was performed using the StepOnePlus™ Real-Time PCR System with *Power* SYBR® Green PCR Master mix (Applied Biosystems) using the following amplification protocol: 2 minutes at 50°C followed by 40 cycles of 15 seconds at 95°C and 1 minute at 60°C. Per reaction 3 µl of diluted cDNA was used and the DNA was amplified using primers for the sequences of interest, listed in table 1. To amplify XBP1 cDNA, PCR was for 32 cycles (95°C for 30 s; 58°C for 30 s; and 72°C for 2 min or 4 min in the final cycle) using XBP1 PCR up and low oligo's with *Taq* DNA polymerase. 398 and 424 bp fragments representing spliced (XBP1s) and unspliced (XBP1u) XBP1, plus a hybrid (XBP1h) migrating as a fragment of approximately 450 bp, were documented after staining 3% agarose gels with ethidium bromide. XBP1h represents a mixture of two hybrid structures. Each structure contains one strand from XBP1s and one strand from XBP1u and is formed in the final annealing PCR step [59]. XBP1h has also been observed by others [60], [61].

### Western blotting

Immunoblot analysis was performed with cell lysates from HEK-cDNA5 and HEK-dnHSF1 cells as described previously [41]. For western blot analysis, polyclonal HSF1 antibody (SPA-901; Stressgen) was used at a 1∶15,000 dilution, HSPA1A antibody 4G4 (ab5444; Abcam) was used at a 1∶5,000 dilution, polyclonal HSPA5 antibody, kindly donated by Prof. Dr. Ineke Braakman, was used at a dilution of 1∶1000, polyclonal DNAJB1 antibody (anti-Hsp40; SPA-400; Stressgen) at a 1∶10,000 dilution, HSPB1 antibody, obtained from Dr. A. Zantema, at a dilution of 1∶400, polyclonal XBP1 antibody [(M-186): sc-7160; Santa Cruz Biotechnology], at a dilution of 1∶200, polyclonal ATF4 antibody [(C-20): sc-200; Santa Cruz Biotechnology], at a dilution of 1∶1000, monoclonal eIF2α antibody was at a 1∶500 dilution, polyclonal phosphorylated eIF2α antibody (E2152; Sigma) was used at a 1∶1,000 dilution, monoclonal γ-tubulin antibody (GTU-88; Abcam) at 1∶1000 dilution and monoclonal β-actin antibody (AC-15, Sigma-Aldrich) at a dilution of 1∶5,000. Blots were incubated with fluorescent secondary antibodies IRDye® 800 CW conjugated goat (polyclonal) Anti-Rabbit IgG and IRDye® 800CW conjugated goat (polyclonal) Anti-Mouse IgG. (926-32211 and 926-32210, respectively; LI-COR Biosciences) according to the manufacturer's instructions and scanned using a LI-COR Odyssey infrared scanner. Signals were quantified using Odyssey version 2.1 software.

## Supporting Information

Figure S1 **The levels of exogenously expressed XBP1s (left panel) or ATF4 (right panel) were determined by western blotting with β-actin as a loading control.** See also legend to Fig. 4A.
(TIF)

Figure S2 **The levels of dnHSF1 and endogenous HSF1 in HEK-pcDNA5 and HEK-dnHSF1 were determined by western blotting.** Cells were harvested after heat shock at the times indicated. Equal amounts of cellular protein were loaded.
(TIF)
