# Supplementary figures and images for: An Atypical Unfolded Protein Response in Heat Shocked Cells
Source: PLoS One. 2011 Aug 10;6(8):e23512. doi: 10.1371/journal.pone.0023512 (PMC3154502; doi:10.1371/journal.pone.0023512)

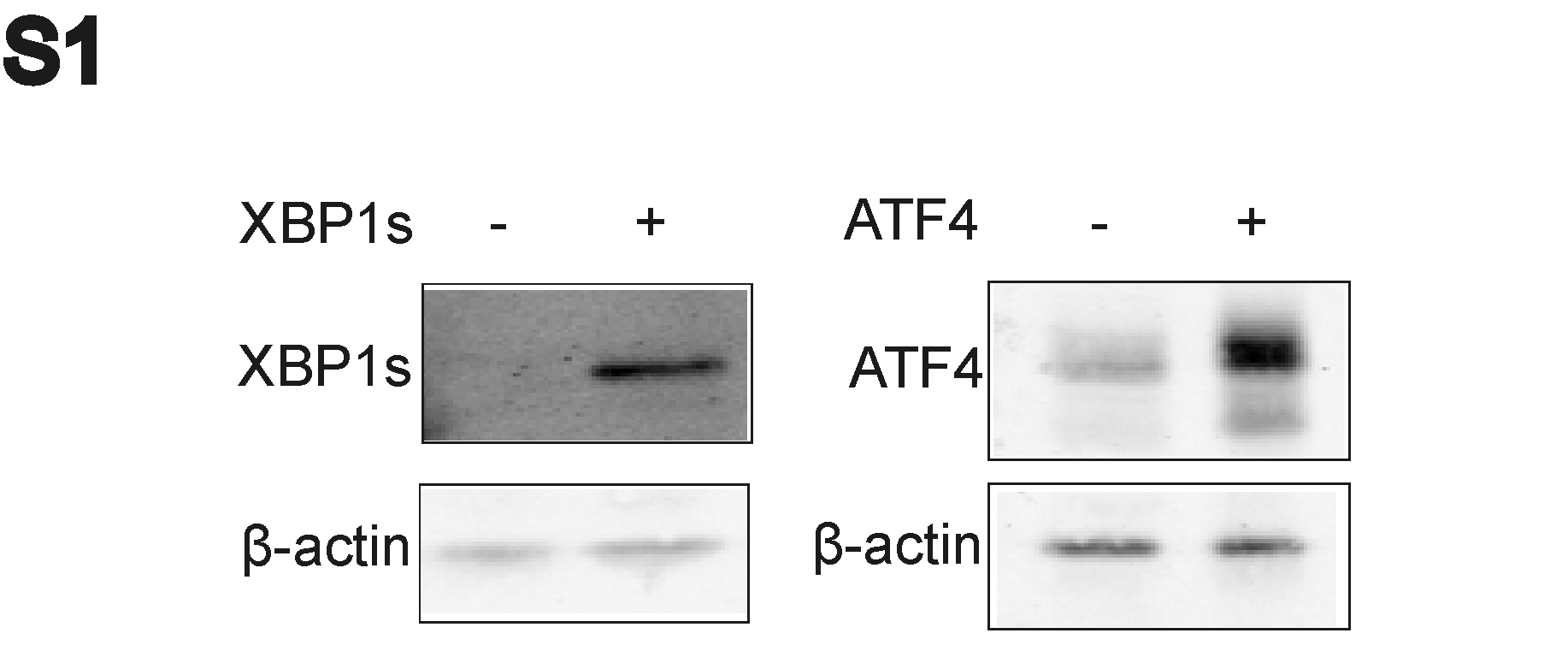

Supplement: Figure S1 — The levels of exogenously expressed XBP1s (left panel) or ATF4 (right panel) were determined by western blotting with β-actin as a loading control. See also legend to Fig. 4A. (TIF) [file pone.0023512.s001.tif]

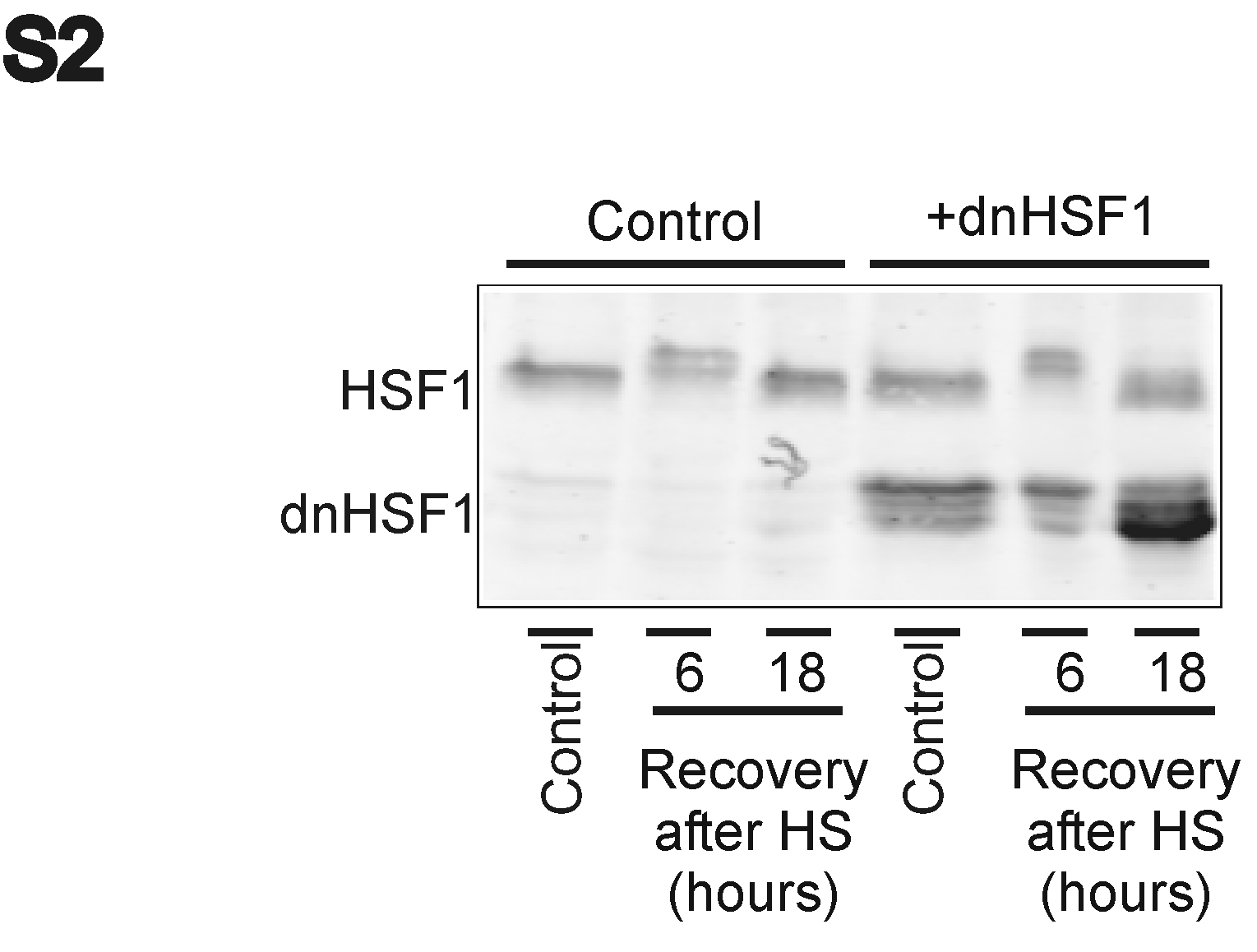

Supplement: Figure S2 — The levels of dnHSF1 and endogenous HSF1 in HEK-pcDNA5 and HEK-dnHSF1 were determined by western blotting. Cells were harvested after heat shock at the times indicated. Equal amounts of cellular protein were loaded. (TIF) [file pone.0023512.s002.tif]
